# Mandibular First Premolar with Three Roots: A Case Report

**Published:** 2012-10-13

**Authors:** Pooja Kakkar, Anant Singh

**Affiliations:** 1. Department of Conservative Dentistry and Endodontics, Sardar Patel Post Graduate Institute of Dental and Medical Sciences, Lucknow, India

**Keywords:** Abnormality, Bicuspid, Dental Pulp Cavity, Root Canal

## Abstract

Anatomy of the root canal system always effects endodontic treatment outcome. Mandibular premolar teeth show extreme variations in root canal morphology. First premolars usually exhibit basic one root and one canal anatomy. The occurrence of three roots in mandibular first premolar has not been commonly reported in literature. This article reports a case of successful nonsurgical endodontic management of mandibular first premolar with three canals and three different apical foramina.

## Introduction

The main objective of endodontic therapy is thorough mechanical and chemical debridement of the entire root canal followed by a three dimensional obturation with an inert filling material and final coronal restoration. Among the major causes of endodontic treatment, failure such as incorrect canal instrumentation, incomplete obturation and untreated major canals, failure to recognize the presence of an additional root canal may result in unsuccessful treatment and may be the origin of acute flare ups during and after treatment [1].

A thorough knowledge of root canal anatomy is therefore a necessity for successful endodontic treatment. Slowey has indicated that due to the variations in canal anatomy, mandibular premolars are the most difficult teeth to treat endodontically; they have a high flare up and failure rate [2].

Root canal morphology of mandibular premolars and differences between first and second premolars have been examined in detail [3][4][5]. In a classic anatomical study, Zillich and Dowson showed the occurrence of three canals in mandibular second premolars to be 0.4% [6]. Literature review of successful clinically reported cases are sparse but also corroborate these findings [7][8][9].

The incidence of number of root canals and apical foramina in mandibular first premolars shows a large variation. Data from anatomical studies report that three rooted mandibular first premolars are rare, about 0.2% [3]. Clinically reported cases showing the presence of three separate roots for the same are few and far between [10][11][12][13][14].

This report presents a case of successful nonsurgical endodontic management of mandibular first premolar with three separate roots using spiral CT.

## Case report

A nineteen year old female patient of Indian descent was referred to the Post Graduate Department of Conservative Dentistry and Endodontics with the chief complaint of intermittent pain over three months in relation to lower left posterior teeth. Patient also complained of episodes of sensitivity to hot foods in the involved tooth. Medical and dental history were non-contributory.

On clinical examination, patient’s oral hygiene was found to be moderate. Deep occlusal carious lesion was observed in tooth # 21 and was tender on percussion. The crown of mandibular first premolar on the contralateral side showed no unusual anatomy in terms of number of cusps and dimension suggestive of any anomaly. Electric pulp test (Sybron Endo, USA) and heat test with a gutta-percha stick gave a lingering response. There was no evidence of swelling or sinus tract.

**Figure 1:**
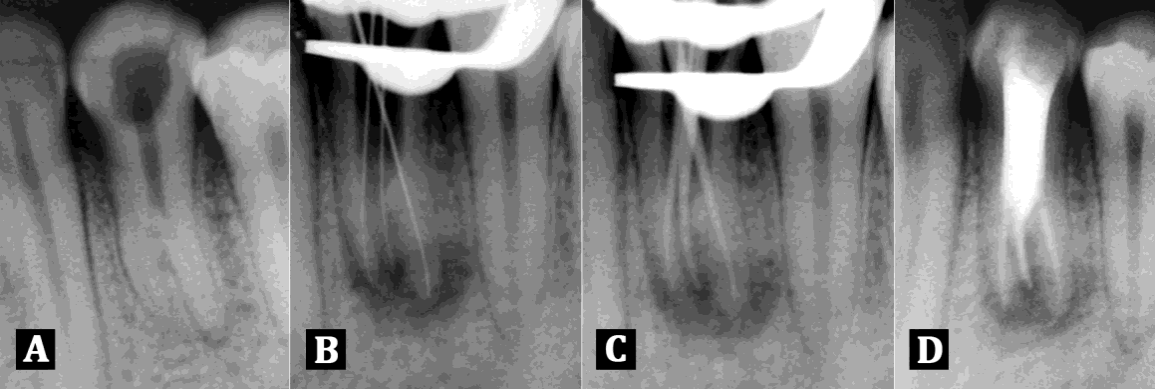
A) Diagnostic radiograph showing three roots inmandibular left first premolar; B) Working length radiograph of three rooted mandibular left first premolar was taken with size 10 K files; C) Master cone radiograph of three rooted mandibular left first premolar was taken with F1 protaper cones; D) Radiograph showing obturation of all the three canals of mandibular left first premolar

Preoperative periapical radiographic examination revealed radiolucency in association with tooth #21 (Figure 1A).

Radiograph also showed the presence of two roots. A second radiograph with more mesial angulation was taken to clearly confirm the presence of a third root. Two roots were found with a shadow of a third root in between the first two.

Based on clinical and radiographic evidences a diagnosis of irreversible pulpitis was made.

Access was gained to the pulp chamber after administration of local anesthesia (2% Lidocaine with1:80,000 adrenaline) under rubber dam isolation. To gain sufficient access to the canals, the conventional access opening was modified into one that was wider mesiodistally. Radiographically, the mid-root diameter appeared to be almost equal to the crown diameter. Tactile examination of the walls of major canals was done with a small precurved pathfinder file (Dentsply, Maillefer, USA) which was advanced slowly down each wall of the major canal, probing for a catch. A slight catch may signify the orifice of an additional canal especially in the case of the buccal and lingual walls because these are the unseen dimensions on the radiograph [9]. Orifice location was difficult as the coronal pulp chamber was unusually long and the separation of roots was from the middle third of the root.

Finally, the three canal orifices were located under magnification using an operating microscope (Zeiss, Oberkochen, Germany) and patency was ascertained with a small size 10 K-file (Dentsply, Maillefer, USA). The working length radiograph was taken (Figure 1B).

Gates Glidden drills were applied (Dentsply, Maillefer, USA) with brushing motion in a crown down fashion to enlarge the orifice to achieve a straight line access. The canals were cleaned and shaped sequentially with ProTaper files (Dentsply, Maillefer, USA), irrigated using 3% sodium hypochlorite and a final rinse of saline. The canals were dried with paper points (Dentsply, Maillefer, USA), cotton was placed in the pulp chamber and Cavite (3M ESPE, St. Paul, MN, USA) was used to close the access cavity. At the second appointment the canals were obturated with F1 ProTaper gutta-percha cones (Dentsply, Maillefer, USA) using AH Plus sealer (Dentsply, Maillefer, USA) (Figure 1C) and (Figure 1D).

**Figure 2:**
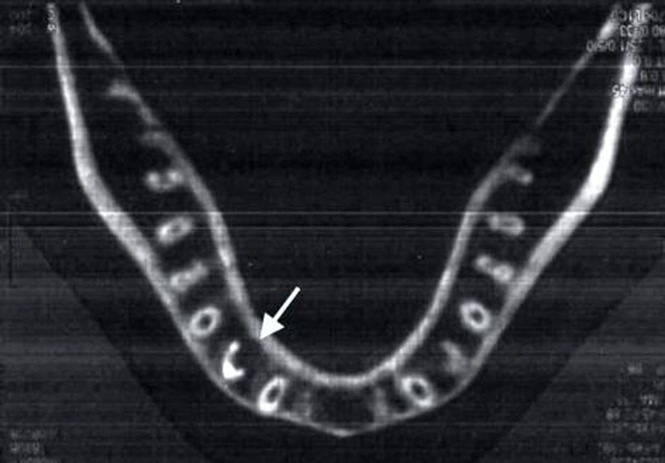
Spiral CT after obturation showing three roots and three obturated root canals in mandibular left first premolar

The access cavity was filled with silver amalgam. To confirm the complex root canal anatomy of the tooth a spiral CT scan (Siemens, Forchheim, Germany) using dental software Dentascan (Figure 2) was planned after obturation and informed consent of the patient was obtained.

## Discussion

Anatomical variations of mandibular premolars are well documented in literature both in terms of anatomic studies and clinically reported cases [3][4][5][6][7][8][10]. Based on race, only one study by Trope et al. showed an increased prevalence of two or more canals in mandibular first premolar in African American patients as opposed to Caucasian American patients [15].

The incidence of mandibular premolars with more than one canal or root is likely to be greater than that reported/found because of hidden images radiographically.

The Washington study which assessed the results of endodontic therapy of mandibular premolars showed that the failure rate in mandibular first premolar as 11.45% [16].

This may be due the extreme variations in root canal morphology of mandibular premolar teeth compared with the standard description of one root and one canal and therefore poses an endodontic challenge to the clinician.

There have been reports of flare-ups in mandibular premolars with associated paresthesia of the inferior alveolar and mental nerves because of missed root canals. The anatomic position of mental foramen and neurovascular structures that pass through the mandible are in close proximity to the apices of mandibular premolars [17].

From a periodontal viewpoint, root configuration is an important factor in the assessment of tooth suitability as a bridge abutment. Multi-rooted premolars with separate roots will offer periodontal support for bridge abutments than similar teeth with roots that converge or present a conical configuration [18].

Moreover, during exodontias, if multi-rooted premolar teeth are rotated during extraction there is an increased likelihood of root fracture [19]. Hence, good quality radiographs are of paramount importance in determining both external and internal root morphology. Two radiographs at 20 degree horizontal angulation should be available for preoperative evaluation of any mandibular premolar. However, radiographs produce only a two dimensional image of a three dimensional object resulting in superimposition of images. Therefore they are of limited value in cases with complex root canal anatomy.

**Figure 3:**
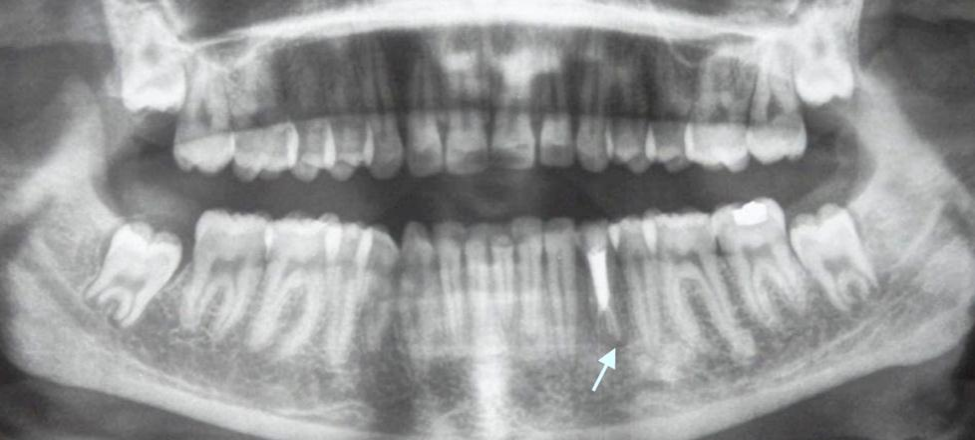
Panoramic view showing obturation of unilateral three rooted mandibular left first premolar

Panoramic view showed this anatomic variation to be unilateral in appearance only (Figure 3).

The advent of 3D imaging such as cone beam tomography and the more recent tuned aperture computed tomography has provided the endodontist with sophisticated diagnostic tools for effective evaluation of root canal morphology that were not available to the clinician before and facilitated interactive image manipulation and enhancement to visualize the area of interest [20]. Spiral CT was taken after obturation which confirmed the presence of three roots as buccal, mesiolingual and distolingual. All the root canals had separate apical foramina. However, in general, the high cost, accessibility and availability to patient and extra radiation as compared to standard radiographic methods makes its routine use limited.

We can conclude that a thorough knowledge of root canal anatomy and its variations, careful interpretation of the radiographs, close clinical inspection of the floor of the chamber and proper modification of access opening along with adequate magnification are essential for successful treatment outcome.

## Conclusions

It is well established that the presence of extra roots and root canals in these teeth may occur far more than one can expect. The clinician should be astute enough to identify the presence of unusual numbers of roots and their morphology.
